# Supplementary figures and images for: An intergenic non-coding RNA promoter required for histone modifications in the human β-globin chromatin domain
Source: PLoS One. 2019 Aug 14;14(8):e0217532. doi: 10.1371/journal.pone.0217532 (PMC6693763; doi:10.1371/journal.pone.0217532)

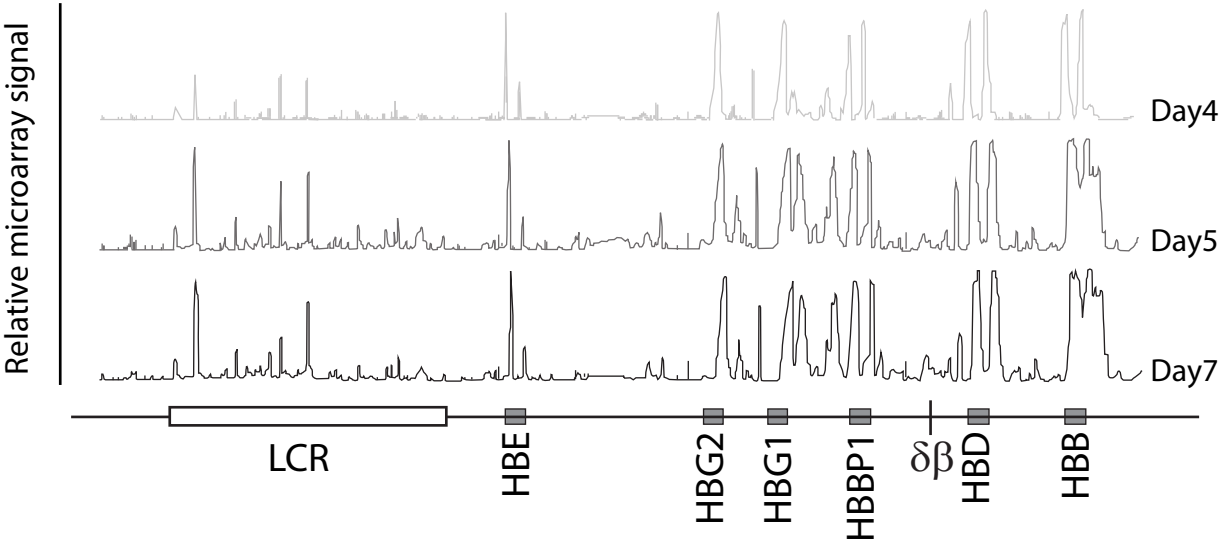

Supplement: S1 Fig — The line below the microarray data is a schematic diagram of the human β-globin locus. Gray boxes represent globin genes; the white box represents the locus control region (LCR); and the vertical line represents the location of the δβ promoter region (see Fig 2A in the manuscript). The graph above the schematic indicates microarray signal intensity for probes of the β-like globin genes (HBE = embryonic ε-globin; HBG2 = fetal Gγ-globin; HBG1 = fetal Aγ-globin; HBBP1 = ψβ-globin pseudogene; HBD = adult δ-globin; HBB = adult β-globin) following labelling and hybridisation of RNA extracted from primary erythroid cells at different phases (day 4 in light grey, day 5 in dark grey and day 7 in black) of phase II culture. The results shown are representative of five biological replicates. (PDF) [file pone.0217532.s001.pdf]
